# Genetic landscape of long noncoding RNA (lncRNAs) in glioblastoma: identification of complex lncRNA regulatory networks and clinically relevant lncRNAs in glioblastoma

**DOI:** 10.18632/oncotarget.25434

**Published:** 2018-07-03

**Authors:** Yashna Paul, Sannu Thomas, Vikas Patil, Naveen Kumar, Baisakhi Mondal, Alangar S. Hegde, Arimappamagan Arivazhagan, Vani Santosh, Kulandaivelu Mahalingam, Kumaravel Somasundaram

**Affiliations:** ^1^ Department of Microbiology and Cell Biology, Indian Institute of Science, 560012 Bangalore, India; ^2^ Sri Satya Sai Institute of Higher Medical Sciences, 560066 Bangalore, India; ^3^ Department of Neurosurgery, National Institute of Mental Health and Neuro Sciences, 560029 Bangalore, India; ^4^ Department of Neuropathology, National Institute of Mental Health and Neuro Sciences, 560029 Bangalore, India; ^5^ Department of Bio-Medical Sciences, School of Biosciences and Technology, VIT University, 632014 Vellore, India

**Keywords:** lncRNA, glioblastoma, ceRNA, ANRIL, CDKN2A-AS1

## Abstract

The major part of the genome that was previously called junk DNA has been shown to be dynamically transcribed to produce non-coding RNAs. Among them, the long non-coding RNAs (lncRNA) play diverse roles in the cellular context and are therefore involved in various diseases like cancer. LncRNA transcript profiling of glioblastoma (*n* = 19) and control brain samples (*n* = 9) identified 2,774 and 5,016 lncRNAs to be upregulated and downregulated in GBMs respectively. Correlation analysis of differentially regulated lncRNAs with mRNA and lncRNA identified several lncRNAs that may potentially regulate many tumor relevant mRNAs and lncRNAs both at nearby locations (*cis*) and far locations (*trans*). Integration of our data set with TCGA GBM RNA-Seq data (*n* = 172) revealed many lncRNAs as a host as well as decoy for many tumor regulated miRNAs. The expression pattern of seven lncRNAs- HOXD-AS2, RP4-792G4.2, CRNDE, ANRIL, RP11-389G6.3, RP11-325122.2 and AC123886.2 was validated by TCGA RNA-Seq data and RT-qPCR. Silencing ANRIL, a GBM upregulated lncRNA, inhibited glioma cell proliferation and colony growth. Cox regression analysis identified several prognostic lncRNAs. An lncRNA risk score derived from five lnRNAs-RP6-99M1.2, SOX21-AS1, CTD-2127H9.1, RP11-375B1.3 and RP3-449M8.9 predicted survival independent of all other markers. Multivariate cox regression analysis involving G-CIMP, IDH1 mutation, MGMT promoter methylation identified lncRNA risk score to be an independent poor predictor of GBM survival. The lncRNA risk score also stratified GBM patients into low and high risk with significant survival difference. Thus our study demonstrates the importance of lncRNA in GBM pathology and underscores the potential possibility of targeting lncRNA for GBM therapy.

## INTRODUCTION

The importance of non-coding RNAs in cellular functions has been widely reported and this in turn indicates their significance in various diseases like cancer. lncRNAs account for a majority of the non-coding transcriptome of the cell and they have been demonstrated to function at the level of both transcriptional and post-transcriptional gene regulation [1]. Ranging from roles in blocking the activity of tumour suppressor genes and inhibiting angiogenesis [2, 3] to functioning as tumour suppressors [4], lncRNAs have important implications in tumour formation and progression. lncRNAs have also been implicated in activating invasion and metastasis [5, 6], raising the stakes on the role of lncRNAs in cancer.

lncRNAs have been shown to interact with RNA, DNA and even proteins [1]. This may be facilitated by their secondary structure or through a sequence-dependent mode. This property aids in the regulatory role they play at the transcriptional and post-transcriptional level. At the transcriptional level, they may act as a molecular signal for transcriptional activity or may guide specific complexes to the site of transcription. The enrichment of RNA Polymerase II at enhancer elements has been shown to be dependent on the transcription of lncRNAs in breast and prostate cancers [7]. This has added merit to the evidence of RNA transcription at enhancer elements and confirmed the production of enhancer associated lncRNAs [8]. In addition, loss-of-function studies have indicated a decreased expression of target genes when their associated lncRNAs are degraded [9]. These reports warranted a study into the effects of the differentially expressed lncRNAs which we have carried out by checking their correlation with mRNAs that were differentially expressed, on a sample to sample basis.

The role of miRNAs in diverse cellular physiological processes and in the cancer scenario is a well-studied field [10]. Among their many roles, miRNAs have also been shown to regulate the expression levels of lncRNAs. A study by Braconi and group in 2011, demonstrated the role of miR-29 in the promoter methylation of MEG3 in hepatocellular carcinoma. The reverse also holds true, with reports of lncRNAs serving as competing endogenous RNAs (ceRNAs) to sequester miRNAs and in turn, affect their mRNA targets [11–13].

While several reports described a genome-wide comprehensive characterization of regulation of lncRNA in many cancers [14–16], there are only few reports of lncRNA role in glioma. Few studies in recent years have reported the role of lncRNAs in glioma development [6, 17, 18]. A recent study using TCGA RNA-Seq data reported the regulation and survival association of lncRNAs in glioma including low grades subtypes and GBMs [19]. Our study, mainly focussed on GBMs, is a microarray based profiling lncRNA and mRNAs simultaneously that enabled us to identify co-regulating lncRNA-mRNA and lncRNA-lncRNA pairs. We also used TCGA RNA-Seq to identify many lncRNAs that serve as host as well as decoy for many tumor regulated miRNAs. Cox proportional regression analysis revealed the survival associated lncRNAs with an lncRNA risk-score developed using most significant lncRNA identified to be an independent predictor of survival in GBM

## RESULTS

### Comprehensive genome analysis of lncRNAs in GBM

**Figure 1:**
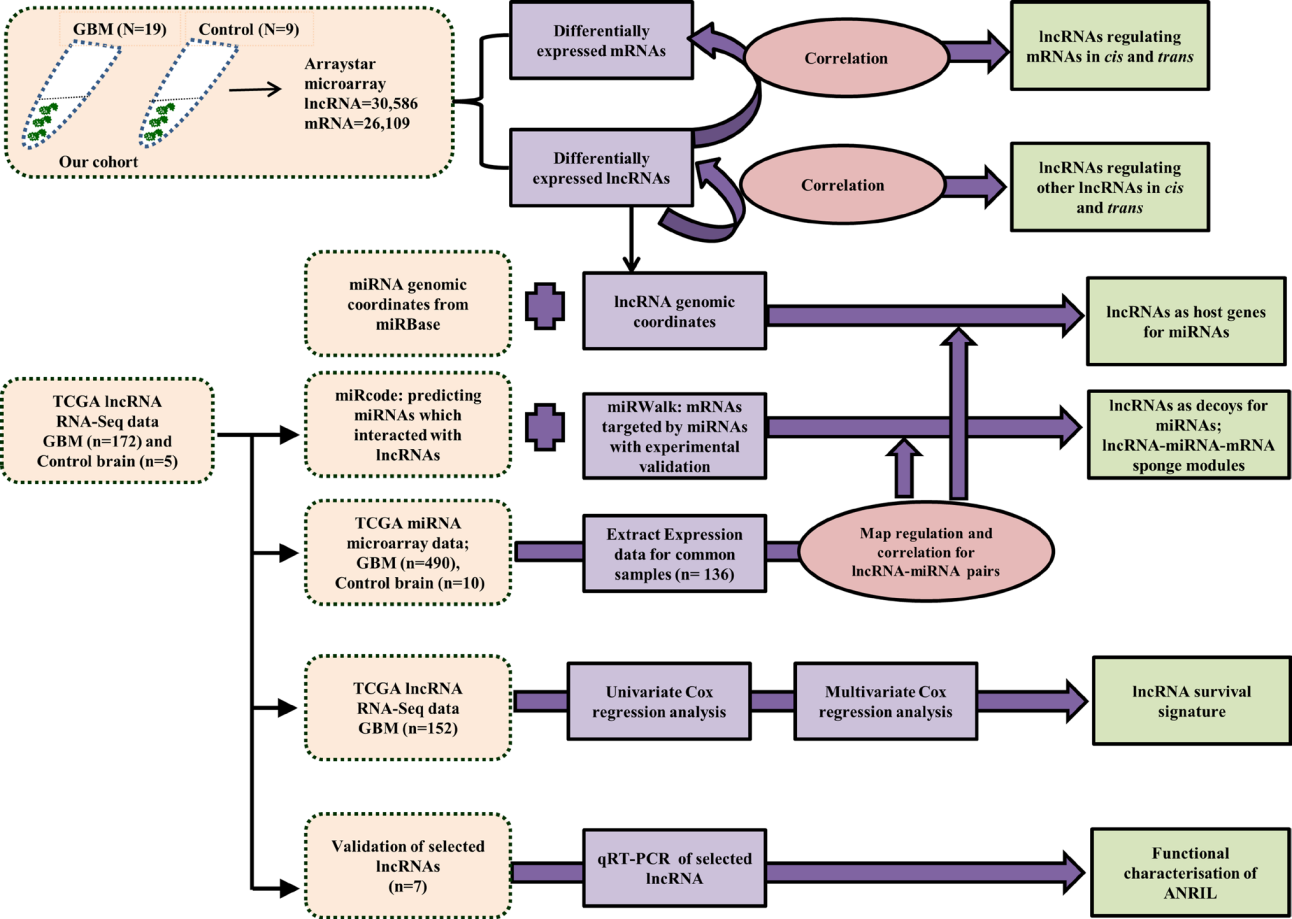
Scheme of the computational analyses carried out in the study The data used for the analysis was either procured from our cohort (lncRNA & mRNA microarray of 19 GBM and 9 control brains), from TCGA (lncRNA RNA-Seq data, miRNA microarray data) or from miRBase (miRNA genomic coordinates, miRNA sequences). The boxes in light pink indicate the source of the data used for the analysis, the purple boxes indicate the input data taken from the different sources, the dark pink boxes indicate the exercises carried out and the green boxes are indicative of the output obtained.

In this study, we have carried out an integrated comprehensive analysis by integrating the expression profile of lncRNAs and mRNA in glioblastoma (GBM) derived from our cohort with lncRNA, miRNA and miRNA expression datasets from TCGA to understand the role of lncRNA mediated gene regulation at the transcriptional and post-transcriptional levels in GBM biology. A schematic diagram of the various types of analyses carried out in this study is shown (Figure 1). This analysis identified clinically relevant lncRNAs with respect to GBM pathobiology. We report here a comprehensive study reporting the microarray based expression profiling of lncRNAs that have been stringently and reliably annotated (*n* = 30,586) in GBM. A microarray that interrogates lncRNA (*n* = 30,586) and mRNA (*n* = 26,109) simultaneously from Arraystar Inc. was used in this study. Comparison of lncRNA expression profile between GBM (*n* = 19) and control brain samples (*n* = 9) identified differentially regulated lncRNAs in GBM. Then the differentially expressed lncRNAs were correlated with differentially regulated mRNAs and lncRNAs to identify the lncRNAs that could potentially regulate mRNAs and lncRNAs in GBM. Next, we investigated their possible post-transcriptional gene regulation through their effect on miRNAs. Coordinates of miRNAs were obtained from miRBase and were mapped to those of lncRNAs in order to derive the lncRNAs that were probable sources of miRNAs. Similarly, lncRNAs that are predicted to target mature miRNAs and thereby probably sequestering them were also derived. From this, lncRNA-miRNA-mRNA sponge modules were identified such that lncRNA could act as sponges in regulating mRNA expression positively by targeting miRNAs. TCGA RNA-Seq data was used to identify prognostic lncRNAs and lncRNA signature was found. The risk score predicted the lncRNA signature to be an independent poor prognostic indicator and it could divide GBMs into high and low risk patients. We have also validated the expression pattern of a number of lncRNAs by RT-qPCR. ANRIL, one of the GBM upregulated lncRNAs was taken up for in-depth functional investigation.

### lncRNAs are highly dysregulated in glioblastoma

**Figure 2:**
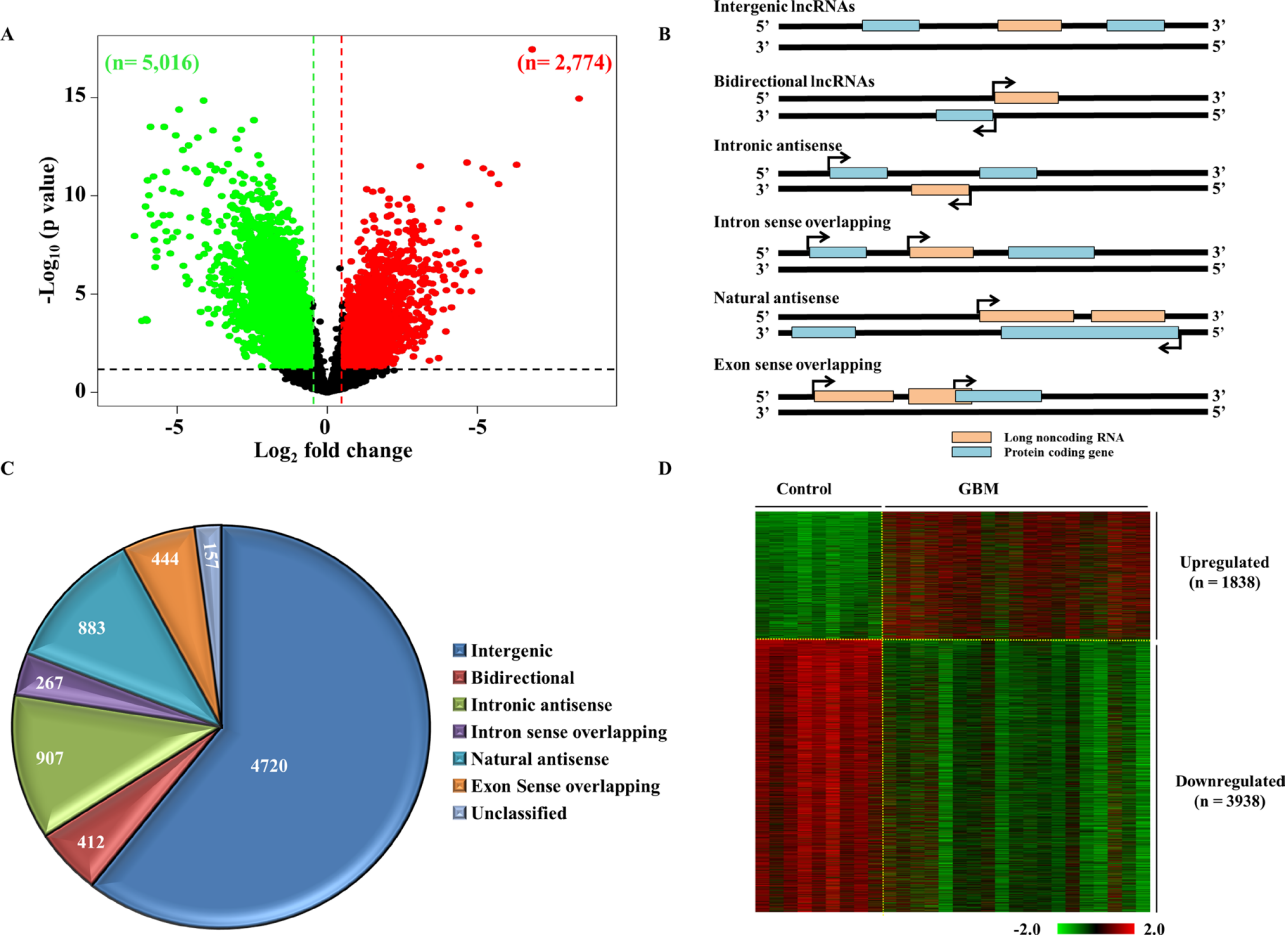
Differential regulation of lncRNAs in GBM (**A**) Volcano plot indicating the upregulated (red), downregulated (green) and unregulated (black) lncRNAs in GBM as compared to control brain. 7,790 lncRNAs were differentially regulated at a difference of ±1.5 fold on the log_2_ scale over the control (*p* < 0.05), which included 2,774 transcripts that were upregulated and 5,016 that were downregulated. (**B**) Schematic representation of the different types of lncRNAs. The lncRNAs (denoted as pink boxes) are classified into 6 groups based on their relative gene positions with respect to nearby mRNA genes (denoted in blue). (**C**) Representation of the classification of dysregulated lncRNAs as a pie chart. The numbers of lncRNAs corresponding to each class are indicated. (**D**) Heat map showing the normalised log_2_ values of the dysregulated lncRNAs genes in GBM over the control samples. Upregulated genes are denoted in red and downregulated genes are shown in green.

To identify the lncRNAs that are dysregulated in GBM, the lncRNA expression profile derived from control brain samples (*n* = 9) and GBM (*n* = 19) was compared (Supplementary Table 1; see methods for a more detailed description). Among the 30,586 lncRNAs transcripts profiled, lncRNAs with a difference of greater than absolute fold change >1.5 in GBM over the control brain samples and a *p* value < 0.05, were taken as significantly dysregulated. There were 2,774 lncRNA transcripts that were upregulated and 5,016 lncRNAs transcripts that were downregulated in GBM (Figure 2A; Supplementary Table 2). Next, we attempted to classify the dysregulated lncRNA into different lncRNA subtypes based on their position and direction of transcription in relation to overlapping or nearby mRNA genes (Figure 2B). The dysregulated lncRNA transcripts were classified into the following sub types: ‘intergenic’, when they lay in the interval between other protein coding genes; ‘intronic antisense’, when they were derived from the antisense to the intron of another gene; ‘intron sense overlapping’, when they were derived from intron of a gene; ‘natural antisense’, when the overlap was with exon(s) of a gene on the antisense strand; ‘exon sense overlapping’, when they overlapped with the exon(s) of a gene; ‘bidirectional’, when they were transcribed to opposite direction to a protein coding gene with start points within 1000 bps. The contribution by each sub type of lncRNAs among dysregulated lncRNAs is shown (Figure 2C and Supplementary Figure 1A). The highest number of dysregulated lncRNAs fell into the intergenic group (*n* = 4,720; 60.59%), indicating that the majority of lncRNAs are regulated as independent transcription units. Next, we removed the exon sense overlapping lncRNAs from the differentially regulated lncRNA to get rid of the possibility of ambiguity that their probes in the microarray might measure both over lapping mRNA and lncRNA. The coordinates of exon sense overlapping lncRNAs that were overlapping entirely with that of the mRNAs were alone removed (Supplementary Figure 1B). This strategy would also aid in avoiding complications which could arise during attempts to validate these lncRNA transcripts in future studies. This step resulted in GBM upregulated and downregulated lncRNAs to 2268 transcripts (1838 lncRNAs) and 4595 transcripts (3938 lncRNAs) respectively (Figure 2D; Supplementary Table 2).

### Potential role of LncRNAs in the regulation of mRNA and lncRNA transcripts

**Figure 3:**
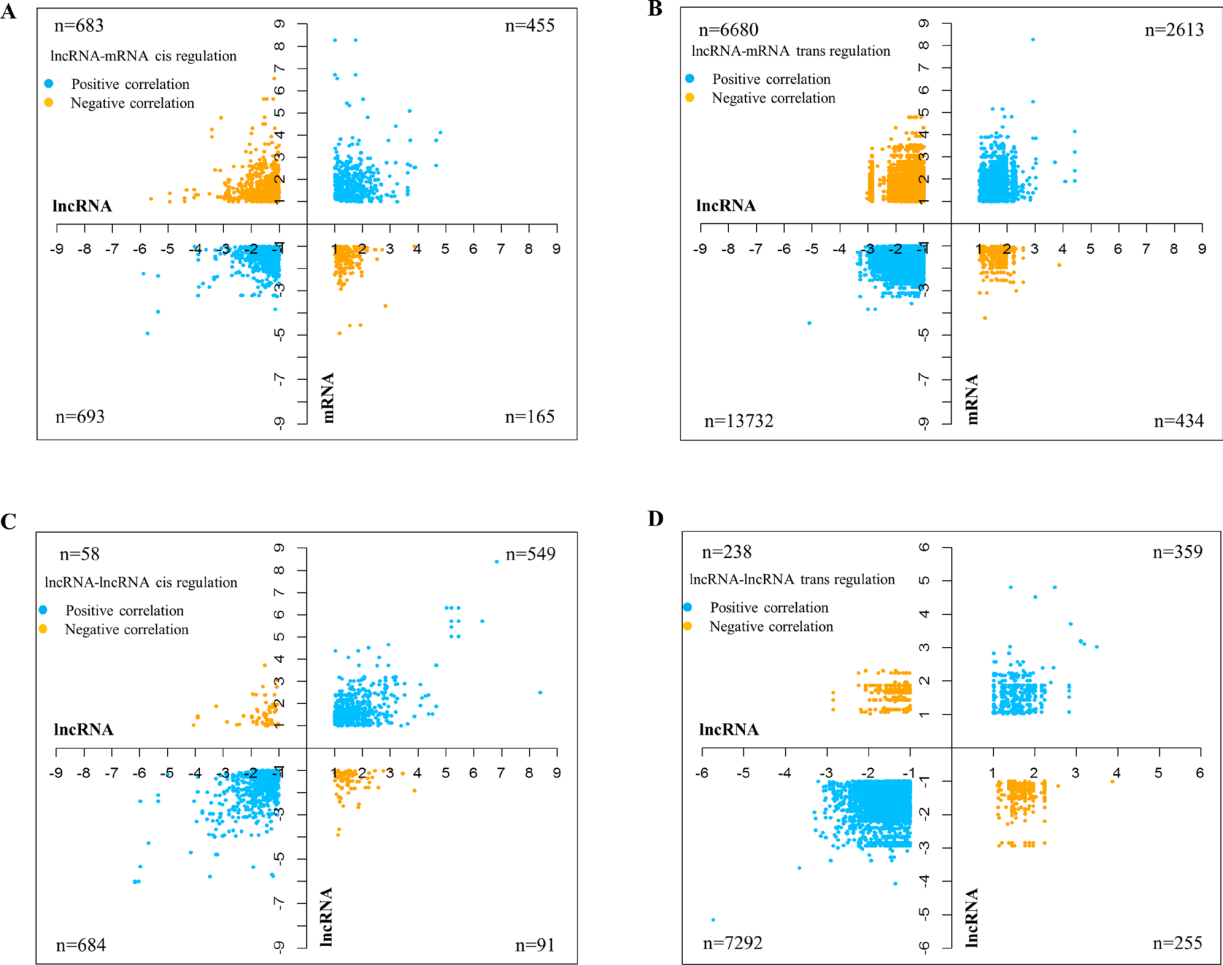
Correlation of lncRNAs with mRNAs and other lncRNA transcripts Graphs showing the correlations between lncRNAs and mRNAs in cis (**A**) and trans (**B**). Each dot represents a lncRNA-mRNA correlating pair. The positive correlations are shown in blue and the yellow dots represent the negative correlations. LncRNA-lncRNA correlations are similarly depicted, with correlations in cis in graph (**C**) and those in trans in graph (**D**). Due to the massive number of correlation pairs obtained for mRNAs in trans, only the pairs having correlation coefficients of 0.9 and above have been represented in (B) and (D). The number of pairs for each of the correlations carried out (*n*), are denoted in the graph.

Correlations between lncRNAs and mRNAs based on their expression profiles have reportedly pointed at co-regulation or functional relatedness [20]. Since the lncRNA microarray from Arraystar also profiled mRNA (*n* = 26,109) expression, we were able to carry out a sample to sample correlation expression analysis between lncRNAs and mRNAs. mRNA transcriptome data analysis revealed that there were 2,806 mRNAs upregulated and 2,270 mRNAs downregulated with a difference of greater than absolute fold change greater than 2 and *p* value < 0.05 in GBM over control brain samples (Supplementary Figure 2A; Supplementary Table 3). Supervised hierarchical clustering led to their partitioning of mRNA transcripts into distinct groups with the control brain samples and GBMs clustering separately (Supplementary Figure 2B). *Cis* regulation by lncRNAs has been demonstrated to occur over long genomic distances, as in the case of the lncRNA Air (IGF2AR) whose regulation spans over a distance of 300 kb [21]. Another study by Li *et al.* in 2013 also indicated the role of lncRNAs in stabilizing enhancer-promoter chromatin looping, thus explaining the distal regulation exhibited by *cis*-acting lncRNAs [22]. Based on the above cited studies, we took a genomic distance of 500 kb upstream and downstream of each of the lncRNA gene coordinate to identify *cis*-regulated genes. The genes which are located beyond ±500 kb upstream and downstream of the lncRNA gene were considered for *trans*-regulation by lncRNA. Then a spearman correlation analysis was carried out between differentially regulated lncRNAs (absolute fold change >2 and *p* value < 0.05) and differentially regulated mRNAs (absolute fold change >2 and *p* value < 0.05). The differentially regulated lncRNAs (absolute fold change > 2 and *p* value < 0.05) were also correlated among themselves to identify the potential regulation of lncRNAs by other lncRNAs (Supplementary Figures 3 and 4). The correlation analysis between lncRNA and mRNA revealed many number of lncRNA-mRNA pairs that act on each other both *cis*- and *trans* fashion. There were 1148 *cis* acting lncRNA-mRNA transcript pairs with positive correlation among them (455 showed upregulation and 693 showed downregulation) and 848 lncRNA-mRNA transcript pairs that are negatively correlated, (165 lncRNA-mRNA pairs wherein upregulated lncRNAs potentially targeting downregulated mRNAs and 683 lncRNA-mRNA pairs wherein downregulated lncRNAs potentially targeting upregulated mRNAs) (Figure 3A; Supplementary Figures 3, 4). This analysis also identified 16,345 *trans* acting lncRNA-mRNA transcript pairs with positive correlation among them (2613 showed upregulation and 13732 showed downregulation) and 7114 lncRNA-mRNA transcript pairs that are negatively correlated, (434 lncRNA-mRNA pairs wherein upregulated lncRNAs potentially targeting downregulated mRNAs and 6680 lncRNA-mRNA pairs wherein downregulated lncRNAs potentially targeting upregulated mRNAs) (Figure 3B; Supplementary Figure 3; Supplementary Table 5). Further to functional significance of regulated lncRNAs that were found to modulate mRNAs in glioma, the list of mRNAs (regulated by lncRNA as per Supplementary Tables 4 and 5) were subjected to pathway and gene ontology analysis by DAVID. As expected, this analysis showed enrichment of terms related to cell proliferation such as “cell cycle”, “DNA replication”, “DNA repair”, “cell division” and pathways such as “Apoptosis” “p53 pathway, “ATM signalling” and “Telomere maintenance” (Supplementary Tables 6 and 7).

To identify lncRNAs that are regulated by other lncRNAs, a correlation analysis was carried out between differentially regulated lncRNAs (absolute fold change >2 and *p* value < 0.05). This analysis revealed many number of lncRNA-lncRNA pairs that act on each other both *cis*- and *trans* fashion. There were 1233 *cis* acting lncRNA-lncRNA transcript pairs with positive correlation among them (549 showed upregulation and 684 showed downregulation) and 149 lncRNA-lncRNA transcript pairs that are negatively correlated, (91 lncRNA-lncRNA pairs wherein upregulated lncRNAs potentially targeting downregulated lncRNAs and 58 lncRNA-lncRNA pairs wherein downregulated lncRNAs potentially targeting upregulated lncRNAs) (Figure 3C; Supplementary Figure 4; Supplementary Table 8). This analysis also identified 7651 *trans* acting lncRNA-lncRNA transcript pairs with positive correlation (359 showed upregulation and 7292 showed downregulation) and 493 lncRNA-lncRNA transcript pairs that are negatively correlated, (255 lncRNA-lncRNA pairs wherein upregulated lncRNAs potentially targeting downregulated lncRNAs and 238 lncRNA-lncRNA pairs wherein downregulated lncRNAs potentially targeting upregulated lncRNAs) (Figure 3D; Supplementary Figure 4; Supplementary Table 9).

### LncRNA regulation of miRNAs as host and sponge

Next, we investigated the potential role of lncRNA in regulating miRNAs. miRNAs have been reported to be transcribed from the exons/introns of lncRNAs as well [23, 24], which adds another dimension to the regulation exhibited by lncRNAs at the post-transcriptional level. The expression of lncRNAs alters the levels of miRNAs, which in turn affect the expression of their target mRNAs [11]. We therefore checked for the presence of dysregulated lncRNAs that served as miRNA hosts. This analysis revealed that a total of 58 differentially regulated lncRNA carried 108 miRNA (see for detail methods; Supplementary Table 10). Further detailed analysis found that there are 13 lncRNA could potentially form the source of 24 miRNA as these lncRNA-miRNA pairs had similar regulation (Table 1). While ten lncRNA-miRNA pairs are found upregulated, three lncRNA-miRNA pairs are down regulated in GBM. There are three lncRNAs that carried more than one miRNA as host lncRNAs. While DLUE2 (Deleted In Lymphocytic Leukemia 2) carried two miRNAs- miR15A and miR16, LINC00478 lncRNA carried three miRNAs-let7c, miR99a and miR125b (Table 1; Supplementary Figure 5). It is interesting to note that the GBM upregulated lncRNA-MIR17HG carried miR17/92 cluster with six miRNAs- miR-17, miR-18a, miR-19a, miR-19b-1, miR-20a and miR-92a-1 (Table 1; Supplementary Figure 5).

**Table 1:** LncRNA genes that behave as hosts or parent genes for miRNAs and miRNA families

Sr. No.	lncRNA name	lncRNA TCGA RNA-seq regulation			miRNA name	miRNA TCGA microarray regulation		TCGA sample wise lncRNA-miRNA correlation	
		log_2_ Fold Change	*p* value	FDR		log_2_ Fold Change	*p* value	coefficient (R)	*p* value
1	LINC00461	1.209	0.001	0.004	hsa-miR-9-3p	0.725	<0.0001	0.380	<0.0001
2	RMST	1.814	0.004	0.017	hsa-miR-135a-5p	1.106	<0.0001	0.414	<0.0001
3	DNM3OS	1.538	0.025	0.077	hsa-miR-199a-3p	0.923	<0.0001	0.464	<0.0001
4	LINC00472	0.837	0.020	0.065	hsa-miR-30a-5p	1.051	<0.0001	0.477	<0.0001
5	MIR155HG	2.647	<0.0001	<0.0001	hsa-miR-155-5p	1.943	<0.0001	0.554	<0.0001
6	BX537900/MIR124-2HG/LINC00966	–3.348	<0.0001	<0.0001	hsa-miR-124-3p	-5.962	<0.0001	0.651	<0.0001
7	LOC100130155/MIR124-2HG/LINC0096	–3.348	<0.0001	<0.0001	hsa-miR-124-3p	-5.962	<0.0001	0.651	<0.0001
8	MIR210HG	1.907	<0.0001	0.001	hsa-miR-210-3p	2.170	<0.0001	0.712	<0.0001
9	MIR7-3HG	–3.299	<0.0001	<0.0001	hsa-miR-7-5p	-3.734	<0.0001	0.736	<0.0001
10	MIR17HG	1.277	0.009	0.033	hsa-miR-92a-3p	1.206	<0.0001	0.231	0.01
					hsa-miR-17-3p	0.811	<0.0001	0.127	0.169
					hsa-miR-17-5p	1.137	<0.0001	0.062	0.4990
					hsa-miR-18a-3p	0.087	<0.0001	0.304	0.0008
					hsa-miR-18a-5p	0.774	<0.0001	0.028	0.755
					hsa-miR-19a-3p	1.031	<0.0001	0.177	0.011
					hsa-miR-19b-3p	1.063	<0.0001	0.161	0.079
					hsa-miR-20a-5p	1.3455	<0.0001	0.063	0.492
11	DLUE2	1.288	<0.0001	<0.0001	hsa-miR-15a-5p	1.466	<0.0001	0.171	0.06
					hsa-miR-16-5p	1.160	<0.0001	0.113	0.22
13	LINC00478	0.514	0.103	0.229	hsa-let-7c-5p	0.314	<0.0001	0.420	<0.0001
					hsa-miR-99a-5p	0.786	<0.0001	0.503	<0.0001
					hsa-miR-125b-5p	-0.133	0.0644	0.29	0.001

**Figure 4:**
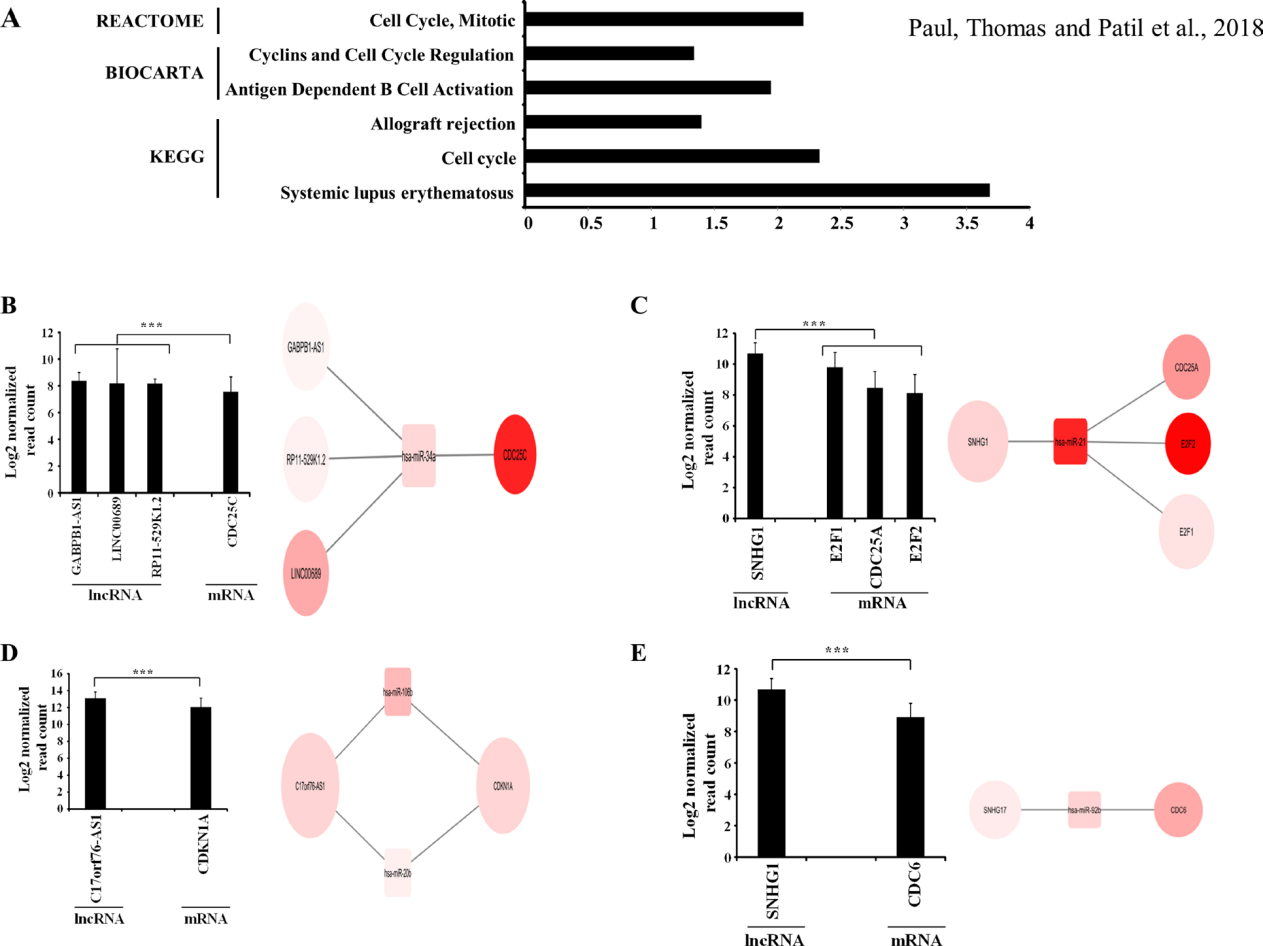
Competing endogenous RNAs (ceRNA) network in GBM (**A**) Significantly enriched pathways in REACTOME, BIOCARTA and KEGG database for ceRNA network genes. (**B**, **C**, **D** and **E**) ceRNA network of four distinct networks related to Cell Cycle pathway in GBM. Bar graph shows the average log_2_ abundance of cell cycle network molecules in GBM samples with standard deviation. Square nodes represent miRNAs and circle nodes represent lncRNAs and mRNAs. The size of the node (lncRNA and mRNA) is proportional to average abundance of the molecule in GBM samples. Intensity of colour represents the log_2_ fold change between GBM samples and control brain samples.

lncRNAs have also been proposed to be more effective competing endogenous RNAs (ceRNA) as compared to mRNAs. We used an integrated analysis with multiple stringent steps involving GBM regulated lncRNAs, miRNAs and mRNAs through target prediction algorithms and experimentally validated miRNA-mRNA pairs to identify lncRNA-miRNA-mRNA sponge module networks (see for details methods). This analysis revealed that there are 409 lncRNA-miRNA-mRNA sponge modules (Supplementary Table 11) that are biologically relevant that the lncRNA in the module is highly likely to work as a sponge such that the target mRNA will be spared from inhibition by miRNAs. These sponge modules have the following characteristics: 1) all molecules- lncRNA, miRNA and mRNA are upregulated in GBM compared to control brain samples, 2) both lncRNA and mRNA have negative significant correlation with miRNA, 3) lncRNA and mRNA have significant positive correlation between them and 4) the abundance of lnRNA transcripts was higher than that of mRNA in GBMs (Supplementary Table 11). Further, to find out important pathways that are regulated by these sponge modules, the unique set of mRNAs (*n* = 140) from this finally selected lncRNA-miRNA-mRNA sponge modules were used for DAVID pathway analysis (Supplementary Table 12). It is interesting to note that the terms related “Cell cycle” was identified by REACTOME, BIOCARTA and KEGG (Figure 4A). The cell cycle related genes E2F1, E2F2, CDKN1A, CDC25A, CDC25C and CDK6 expression appears to be regulated through several lncRNA sponges through the regulation of common miRNA(s) (Figure 4B–4E). Surprisingly, we also found CD28, IL10 and FAS related to activation of T and B lymphocytes, are upregulated and their high levels appears to be regulated by several sponge lncRNAs (Supplementary Figure 6A–6C). Thus, we have identified several lncRNA sponge molecules that appear play important role in regulating many mRNAs through their impact on miRNAs.

### CDKN2B-AS1 or antisense noncoding RNA in the INK4 locus (ANRIL) is highly expressed in GBM but unlikely to be the repressor of INK4 locus

**Figure 5:**
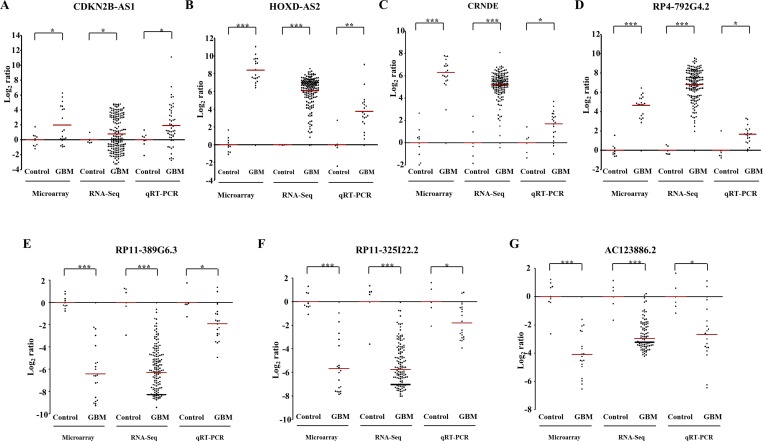
Validation of differentially regulated lncRNAs by RNA sequencing and real time qPCR The expression of lncRNAs in TCGA cohort (RNA sequencing) and our cohort (RT-qPCR) as compared to microarray data (our cohort), has been depicted as scatter plots for CDKN2B-AS1 (**A**), HOXD-AS2 (**B**), CRNDE (**C**), RP4-792G4.2 (**D**), RP11-389G6.3 (**E**), RP11-325I22.2 (**F**) and RP11-325I22.2 (**G**). Each dot represents the data derived from one sample. For each sample, fold change in expression is calculated over its average expression in control brain tissue. Our cohort was a subset of the samples subjected to microarray analysis. The *p* values have been represented by ^*, **^ and ^***^ which denotes values of *p* < 0.05, *p* < 0.01and *p* < 0.001 respectively.

**Figure 6:**
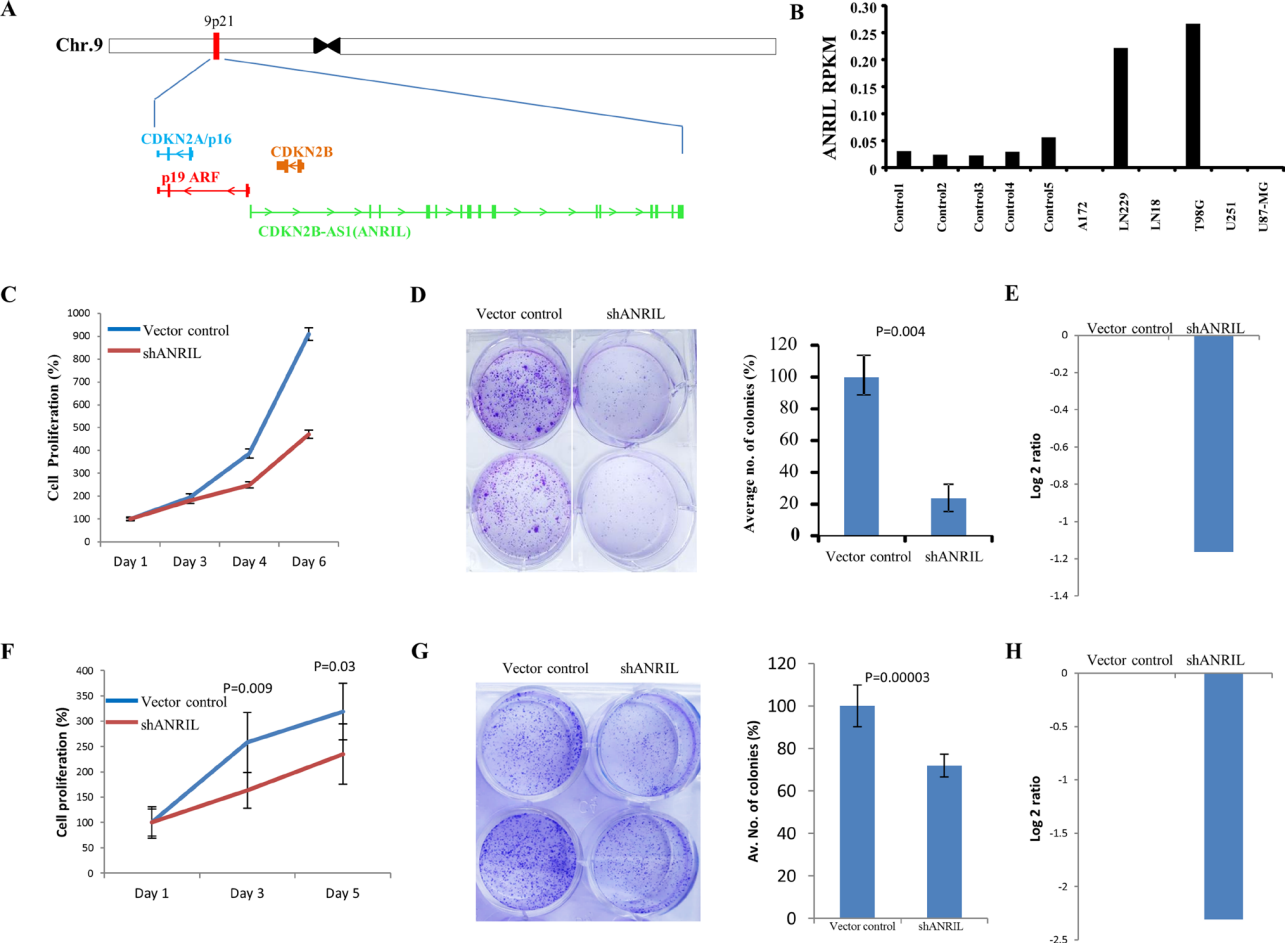
ANRIL is upregulated in GBM and its downregulation results in the inhibition of proliferation (**A**) A snapshot depicting the genomic location of ANRIL gene in the intronic anti-sense region of *CDKN2B* gene. (**B**) Column plot indicating the expression of ANRIL (log_2_ ratio) across different glioma cell lines compared to control brain tissue. (**C**) LN229 cells were transfected with vector control or shANRIL, plated for the proliferation assay and the relative proliferation was quantified by MTT assay. (**D**) LN229 cells were transfected with vector control or shANRIL. The cells were then plated in duplicates in a 6-well plate. After 2 weeks of plating, the colonies were stained with crystal violet, photographed (left) and counted (right). % colony density ± sd is plotted. (**E**) Log_2_ transformed levels of ANRIL transcript upon 48 hrs of shRNA knockdown in LN229 cell line. (**F**) T98G cells were transfected with vector control or shANRIL, plated for the proliferation assay and the relative proliferation was quantified by trypan blue assay. (**G**) T98G cells were transfected with vector control or shANRIL. The cells were then plated in duplicates in a 6-well plate. After 2 weeks of plating, the colonies were stained with crystal violet, photographed (left) and counted (right). % colony density ± sd is plotted. (**H**) Log_2_ transformed levels of ANRIL transcript upon 48 hrs of shRNA knockdown in T98G cell line.

Next, we have taken seven differentially regulated lncRNAs for validation. Four upregulated lncRNAs (CDKN2B-AS2, HOXD-AS2, CRNDE and RP4-792G4.2) and three downregulated lncRNAs (RP11-389G6.3, RP11-325122.2 and AC123886.2) as per microarray data also showed similar regulation in TCGA RNA-Seq data and RT-qPCR data generated from the lab cohort (Figure 5A–5G). The lncRNA ANRIL (Antisense noncoding RNA in the INK4 locus), which is transcribed antisense to the INK4b-ARF-INK4a locus (Figure 6A), was considered for functional characterization with respect to glioma biology. We found the ANRIL transcript showed varying levels in glioma cell lines and silencing ANRIL in LN229 and T98G glioma cell lines inhibited cell proliferation and colony formation (Figure 6B–6H). INK4b/ARF/INK4a locus encodes two cyclin-dependent kinase inhibitors CDKN2B (p15) and CDKN2A (p16) as well as p19ARF which binds to MDM2 and promotes its degradation resulting in the p53 activation [25]. While the INK4b/ARF/INK4a locus is shown to undergo deletion in GBM [26], epigenetic transcriptional repression of the locus by a repressive complex containing ANRIL and CBX7 has also been shown in prostate cancer [27]. We found ANRIL transcripts to be upregulated in GBM (Supplementary Figure 7A). Next, when we analysed the GBM samples divided into two groups on the basis of the presence of homozygous deletion in INK4b/ARF/INK4a locus, we found some interesting results. In contrast to entire cohort of GBM samples, we found a near significant or significant downregulation of ANRIL, CDKN2A and CDKN2B in GBMs with homozygous INK4a/INK4b co-deletion compared to that of control brain samples (Supplementary Figure 7A–7C). We also found a positive significant correlation between transcript levels of ANRIL and CDKN2A as well as CDKN2B in GBMs with homozygous deletion of the INK4b/ARF/INK4a locus (Supplementary Figure 7E and 7F). These results signify co-deletion of all three genes in GBMs with homozygous deletion of the INK4b/ARF/INK4a locus. The analysis of the GBM sub group with no copy number alterations revealed that the upregulation of ANRIL is largely restricted to GBMs with no copy number alterations in INK4b/ARF/INK4a locus (Supplementary Figure 7A). We also found a significant positive correlation between ANRIL and CDKN2A as well as CDKN2B in GBMs with no copy number aberrations (Supplementary Figure 7G and 7H). Moreover, CBX7 is down regulated in GBMs regardless of the deletion status of INK4b/ARF/INK4a locus (Supplementary Figure 7D). These results demonstrate that INK4b/ARF/INK4a locus is not repressed by ANRIL in GBMs possibly due to the downregulation of CBX7. Thus, we conclude that ANRIL is upregulated in GBM and is an oncogenic lnRNA in GBM. Further, unlike what is shown in prostate cancer [27], ANRIL is not likely to be the repressor of INK4b/ARF/INK4a locus in GBM.

### LncRNA expression correlates with survival of GBM patients

**Figure 7:**
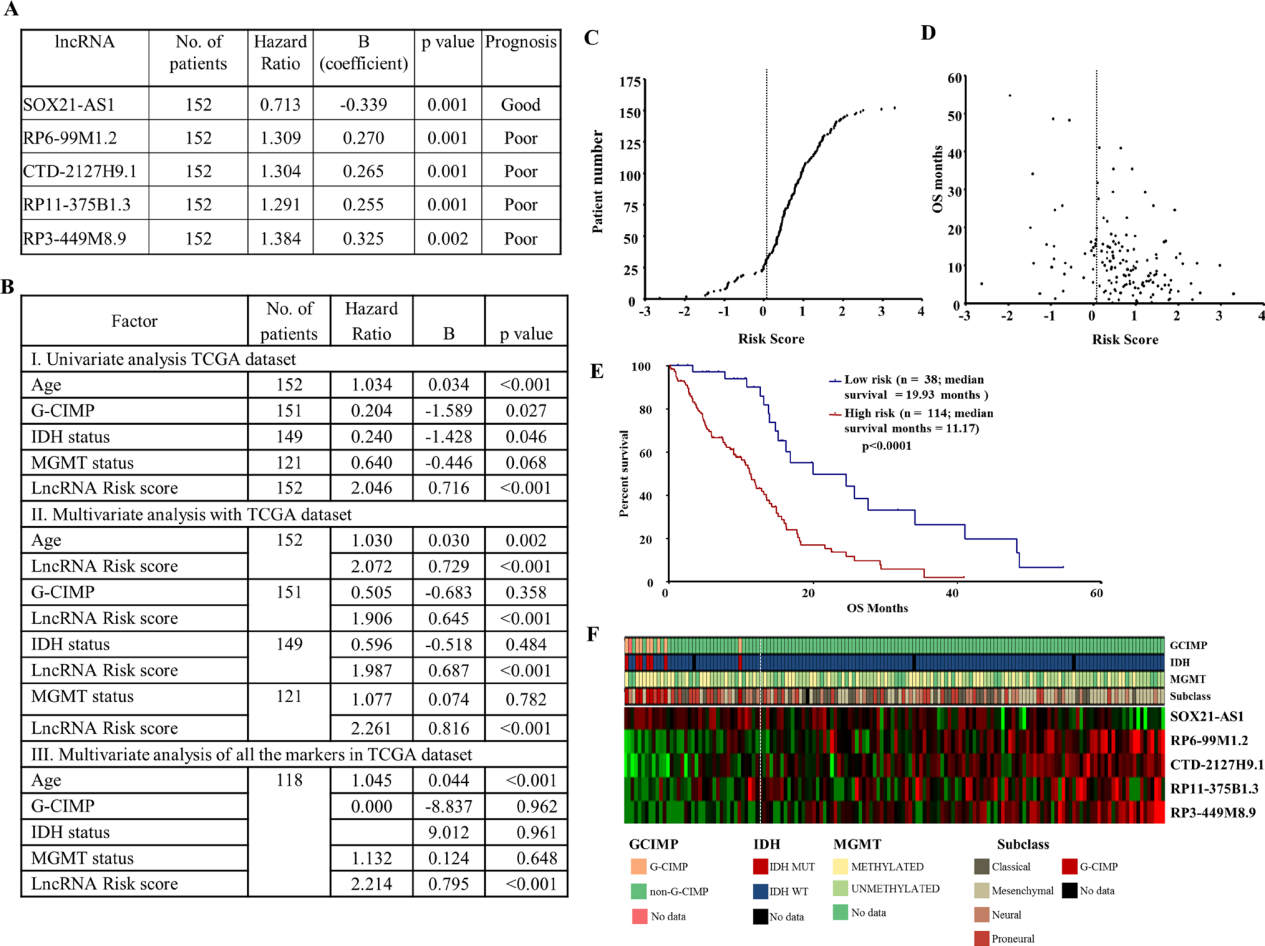
Prognostic role of lncRNA signature in GBM (**A**) Univarate Cox proportional hazard regression analysis of five lncRNAs using TCGA RNA-Seq. data. (**B**) Univariate and multivariate Cox proportional hazard regression analysis of lncRNA signature risk score with age, G-CIMP methylation status, IDH mutation and MGMT promoter methylation status. (**C**) lncRNA risk score distribution of the GBM patients from TCGA RNA-Seq cohort. (**D**) Patient survival status along with risk score from TCGA RNA sequencing cohort. (**E**) Kaplan–Meier survival estimates overall survival of GBM patients according to the lncRNA expression signature. (**F**) Heat map of five lncRNAs expression profiles of GBM patients; rows represent lncRNAs, and columns represent patients. The dotted white line represents the lncRNA signature cut-off dividing patients into low-risk and high-risk groups.

To identify the lncRNAs that correlate to GBM survival, TCGA RNA-Seq data was used (*n* = 152). We defined the lncRNAs based on the manually annotated lncRNA genes from Gencode v19, which included 13,853 lncRNAs [28]. Additional filters were used to use the lncRNAs, wherein the quantitation is reliable (see detail in methods). A total of 4840 lncRNA corresponding to intergenic, intron sense overlapping and processed transcripts and lncRNAs having at least 1 read in ≥ 30% of TCGA RNA-Seq samples were considered for univariate Cox regression analysis. Out of 4840 lncRNAs, 309 showed a significant correlation with survival (*p* value < 0.05) (Supplementary Table 13). Using stringent criteria, the top five lncRNAs were chosen for further analysis. While SOX21-AS1 showed good prognosis, the other four lncRNAs- RP6-99M1.2, CTD-2127H9.1, RP11-375B1.3 and RP3-449M8.9 showed poor prognosis (Figure 7A). An lncRNA risk score was made combining the survival prediction capability of all five lncRNAs by using a risk score formula (see for details methods). The lncRNA risk score predicted survival significantly in a univariate analysis as well as a multivariate analysis involving age, G-CIMP, IDH1 mutation, MGMT promoter methylation (Figure 7B). The distribution of lncRNA risk score and a comparison of risk score with patient survival are shown (Figure 7C and 7D). Further, lncRNA risk score stratified GBMs into low- and high-risk groups with significant difference in patient survival (median survival: 19.93 vs 11.17 months; Figure 7E). The expression pattern of five lncRNA between the low- and high-risk groups is shown (Figure 7F). SOX21-AS1 appears to act like a protective lncRNA as it expression was found to be higher in low risk group. In contrast, the other four lncRNAs appear to be risky lncRNAs as their expression is more in high-risk group. While the G-CIMP+ and IDH1 mutation samples entirely belong to low-risk group, the MGMT promoter methylation status and gene expression sub types appear to be equally distributed between low- and high-risk groups.

## DISCUSSION

Aberrant transcription, a hallmark of cancer, is responsible for the dysregulation of transcripts in the cell. The differential expression of lncRNAs in cancer is implicative of their role in the malignancy [14–16]. However, there also exists the possibility that their altered expression could be an outcome of the disease, with no consequential role in its malignancy. Hence using lncRNA expression profiling, we have sought to identify lncRNAs whose aberrant expressions might translate to functions, thereby narrowing down on those with physiological relevance. Based on the known functions of lncRNAs, we used attributes such as their sequence and expression features to computationally narrow down on the prospective roles of the differentially expressed lncRNAs.

Microarray profiling studies of lncRNA genes in glioma has led to the discovery of several transcripts that have been used for subtype classification [29–31] and for the development of molecular signatures [32]. However, most of these studies have been conducted with the utilization of mined data from existing arrays that have coincidental representation of lncRNA probes. This approach faces the limitation of receiving an underrepresentation of the dysregulated lncRNAs in the disease. Our study that has employed the analysis of over 30,000 lncRNAs, therefore offers a highly comprehensive estimation of the long noncoding RNAs whose expressions are disrupted in GBM. Further, it is noteworthy that we have included a larger cohort for the study, comprising of 19 GBM patient samples, as compared to other studies [33, 34]. We identified a total of 7,790 lncRNA transcripts of which 2,774 transcripts were upregulated and 5,016 were downregulated. The presence of several lncRNAs like CRNDE, HOTAIRM1 and H19 among the upregulated transcripts, were in concordance with earlier reports on their oncogenic role in glioma [6, 30, 33, 35]. Similarly MEG3, a lncRNA with tumour suppressor properties [17], was downregulated in our microarray results. The dysregulated lncRNAs could segregate the control brain samples from the diseased ones, thus indicating their potential prominence in the disease. The vast numbers of dysregulated lncRNA transcripts which ran into thousands, prompted us to subject them to further analysis that would reveal their other significant functional attributes.

The reported mechanisms of lncRNA function majorly involve their regulation of the expression of genes, that may be either proximal to the lncRNA gene or may even be on a different chromosome. However, proximity to a gene need not render the lncRNA a regulator. In addition, regulation of distant genes has been reported to be dependent on their structure and not sequence, as in the case of the lncRNA MEG3 [36]. Therefore, neither the proximity to a gene nor its sequence similarity can implicate the targets of its regulation. We hence used a ‘guilt by association’ strategy [37] and carried out lncRNA-mRNA coexpression analysis on a sample to sample basis. The genomic distance of 500 kb we chose for the estimation of the regulations in *cis*, was on the basis of several reports on enhancer effects over a broader genomic region as in the case of HOTTIP, ecCEBPA, Mistral and Air [9, 21, 38]. Several hundred of lncRNA-mRNA pairs, with correlation between them both positively and negatively, were obtained in *cis* and *trans* as well. It is worth noting several mRNAs coding for proteins relevant to transformation including tumor suppressors, oncogenes, kinases, pro and anti apoptotic proteins. To the best of our knowledge, our analysis on the co-regulation of lncRNAs is the first comprehensive analysis of its kind till date. These correlations would give insight into additional layers of regulation of the lncRNA-mRNA association network.

The regulation exerted by miRNAs in the cell is complex and profound due to the propensity of a single miRNA to regulate several transcripts. Their influence therefore extends to the various biological processes in the cell and consequently to tumour progression. There is significant crosstalk between miRNAs, lncRNAs and mRNAs in the cell, which when depicted as networks, is highly complex. The roles of lncRNAs in this network majorly include their ability to serve either as a source of miRNAs or as a sequester of the miRNAs. H19 has been reported to be the source of miR-675, which targets Insulin growth factor receptor [39]. By mapping the coordinates of the miRNAs to those of dysregulated lncRNAs, we could identify the lncRNAs that harboured miRNAs. From the several lncRNAs obtained as potential miRNA hosts, we could demonstrate that differential regulation of lncRNA as the reason for differential regulation of miRNA. The upregulated MIR17HG gives rise to the miR 17/92 cluster, which is well studied in cancer and in glioblastoma [40] In the next exercise, we obtained several lncRNAs that showed complementarity with the mature sequence of miRNAs. Their opposite regulation is indicative of their potential role as sponges for the miRNAs [11, 41]. We were able to carry out extensive analysis to identify physiologically relevant lncRNA-miRNA-mRNA sponges wherein the lncRNAs are more likely to work as sponges in titrating miRNAs such that target mRNAs are spared from miRNA effects. It is interesting to note that a set of genes related to cell cycle and antigen presentation in particular are regulated by lncRNA sponges.

Several lncRNAs reportedly having crucial roles in cancer, including HOTAIR, MALAT1 and H19, have been reported to be linked to survival [5, 42, 43]. This encouraged us to look into the propensity of the dysregulated lncRNAs to function as prognostic tools in GBM. This analysis revealed several lncRNAs could act as poor or good prognostic markers. Further, we demonstrate that a lncRNA rick-score based on top five lncRNAs is an independent predictor of survival in GBM. We were able to stratify GBMs into low and high-risk groups with significant difference in the survival.

The lncRNA ANRIL has been shown to be upregulated and repress INK4b-ARF-INK4a locus epigenetically by recruiting CBX7 in prostate cancer [27]. While we found ANRIL is upregulated in GBM overall, we found that it is co-deleted in GBMs with homozygous deletion of INK4b-ARF-INK4a locus. In GBMs with no copy number aberration in INK4b-ARF-INK4a locus, we found upregulation of all genes that are present in the locus -ANRIL, CDKN2A and CDKN2B thus signifying the absence of ANRIL-mediated repression of INK4b-ARF-INK4a locus in GBM. On possible reason for the absence of repression of INK4b-ARF-INK4a locus by ANRIL is the downregulation of the co-repressor molecule CBX7 in GBMs. Thus our results demonstrate that ANRIL is upregulated in GBM and is an oncogeneic lnRNA in GBM. Further, unlike what is shown in prostate cancer [27], ANRIL is not likely to be the repressor of INK4b/ARF/INK4a locus in GBM.

This unique *in-silico* functional characterization of lncRNAs provides us the very first comprehensive glimpse of lncRNA regulatory effects in GBM. This study can form the basis and provide direction for several future endeavours that aim to dissect the layer of regulation exerted by lncRNAs in GBM. lncRNAs have been shown to function as therapeutic targets in many diseases. This is of particular importance in cancers like GBM wherein clinicians face difficulty in devising effective treatment strategies. Exploring the functional relevance of lncRNAs in GBM will help give better insights into the deregulated pathways of this disease.

## MATERIALS AND METHODS

### Tumor samples and clinical details

Tumor samples were collected from patients who were operated at National Institute of Mental Health and Neurosciences (NIMHANS) and Sri Satya Sai Institute of Higher Medical Sciences (SSSIHMS), Bangalore, India. A portion of the anterior temporal cortex resected during surgery for drug resistant epilepsy patients served as control brain sample. The study was scrutinized and approved by the ethics committee of the two clinical centres, NIMHANS and SSSIHMS, and patient consent was taken before initiation of the study as per Institute Ethical Committee guidelines and approval. We used a total of 19 GBM patient samples and 9 control brain tissue samples for this study. The histology was confirmed as GBM by the neuropathologist as per WHO 2007 classification scheme.

### Cell lines and plasmid

The glioma cell lines (A172, LN229, LN18, T98G, U251 and U87-MG) used in this study were obtained from Sigma Aldrich, Saint Louis, Missouri, USA. These cells were grown in Dulbeccos modified Eagle’s medium (DMEM) supplemented with 10% FBS. The cells were maintained at 37° C and 5% CO_2._ Retro-virus based shRNA construct for ANRIL (pSR_ANRIL) was a kind gift from Dr Yojiro Kotake, Kindai University, Japan [3]. The control retroviral vector (pSR_SCR) used was obtained from Addgene, USA.

### Tissue RNA isolation

Total RNA was extracted from Glioblastoma (GBM) and control brain tissue samples. Briefly, the tissue was subjected to homogenisation in the presence of trizol reagent. Chloroform was added and the tubes were vortexed vigorously followed by centrifugation at 14,000 rpm for 30 min. Isopropanol was used to precipitate the RNA from the aqueous phase. The pellet was washed with 70% ethanol and dissolved in triple autoclaved distilled water. RNA quantity and integrity was determined by nanodrop and by denaturing agarose gel electrophoresis.

### Arraystar microarray sample preparation and microarray

Arraystar Human lncRNA Microarray V3.0 was used for the global profiling of human lncRNAs and protein-coding transcripts. About 30,586 lncRNAs and 26,109 coding transcripts can be detected by this lncRNA microarray.

### RNA labelling and array hybridization

Sample labelling and array hybridization were performed according to the Agilent One-Color Microarray-Based Gene Expression Analysis protocol (Agilent Technology) with minor modifications. Briefly, mRNA was purified from total RNA after removal of rRNA (mRNA-ONLY™ Eukaryotic mRNA Isolation Kit, Epicentre). Then, each sample was amplified and transcribed into fluorescent cRNA along the entire length of the transcripts without 3’ bias utilizing a random priming method (Arraystar Flash RNA Labeling Kit, Arraystar). The labelled cRNAs were purified by RNeasy Mini Kit (Qiagen). The concentration and specific activity of the labelled cRNAs (pmol Cy3/μg cRNA) were measured by NanoDrop ND-1000. 1 μg of each labelled cRNA was fragmented by adding 5 μl 10 × Blocking Agent and 1 μl of 25 × Fragmentation Buffer, then heated the mixture at 60° C for 30 min, finally 25 μl 2 × GE Hybridization buffer was added to dilute the labelled cRNA. 50 μl of hybridization solution was dispensed into the gasket slide and assembled to the lncRNA expression microarray slide. The slides were incubated for 17 hours at 65° C in an Agilent Hybridization Oven. The hybridized arrays were washed, fixed and scanned with using the Agilent DNA Microarray Scanner (part number G2505C).

### Microarray data analysis

Agilent Feature Extraction software (version 11.0.1.1) was used to analyze acquired array images. Quantile normalization and subsequent data processing were performed with using the GeneSpring GX v12.1 software package (Agilent Technologies). After quantile normalization of the raw data, lncRNAs and mRNAs that at least 7 out of 28 samples have flags in Present or Marginal (“All Targets Value”) were chosen for further data analysis. Differentially expressed lncRNAs were identified by calculating student *t* test between GBM samples and control brain samples (Fold Change ≥ 2.0, *p* value < 0.05).

### TCGA RNA sequencing analysis

We obtained raw RNA sequencing data for GBM samples from TCGA. The whole RNA sequencing data was aligned using PRADA tool [44]. Duplicate removal was carried out using Picard 1.73 [45]. lncRNAs were annotated as per Gencode Version 19 annotation file [28]. The RNA-seq reads were counted over gene exons using HtSeq [46]. We used the DESeq2 size factor correction to account for differences in sequencing depth between the samples [47].

### lncRNA survival analysis

The expression level of each lncRNA was used to check there correlation with survival. The 152 GBM samples (TCGA RNA-seq cohort) for which survival information were available were used for univariate Cox regression analysis using survival package of R (version 3.2.3). SPSS version 19.0 was used for multivariate analysis. Kaplan Meier survival analysis was performed using GraphPad Prism 5.0 version for Windows (GraphPad Software, San Diego, California USA, www.graphpad.com). The risk score for the signature was calculated using the following formula:

Risk score of a sample = ∑ (cox regression coefficient of a particular lncRNA X log_2_ value of expression of lncRNA).

### Integrative analysis of lncRNAs in GBM

#### LncRNAs having enhancer like functions that regulate mRNAs in *cis* and *trans*

Sample wise Spearman correlation was performed between all the differentially expressed lncRNAs (*n* = 4,289) and differentially expressed mRNAs (*n* = 5,076) for 19 GBM samples (our cohort) using R software (version 3.1.0). This analysis resulted in 21,770,964 lncRNA-mRNA pairs. lncRNA-mRNA pairs were divided into two groups (a) *cis* pairs (mRNAs that lie 500 kb within upstream and downstream of the lncRNA coordinates) and (b) *trans* (mRNAs that lie >500 kb upstream and downstream of the lncRNA coordinates). Out of 10,968 lncRNA-mRNA *cis* pairs, 2,674 lncRNA-mRNA *cis* pairs were significantly correlated (*p* value < 0.05). Since the lncRNA-mRNA *trans* pairs were large in number (*n* = 21,759,996), we used stringent cut off (*p* value < 0.05 and |r| >0.9). We found 4,679,124 lncRNA-mRNA *trans* pairs were significantly (*p* value < 0.05) correlated. Out of these pairs 24,631 lncRNA-mRNA *trans* pairs were having absolute Spearman correlation coefficient greater than 0.9 (Supplementary Figure 3).

#### LncRNAs regulating lncRNAs

Sample wise Spearman correlation was performed for all the differentially expressed lncRNAs (*n* = 4,289) for 19 GBM samples (our cohort) using R software (version 3.1.0). From this analysis we found a total of 18,395,521 lncRNA-lncRNA pairs. Correlation analysis of an lncRNA with itself would yield *R* value of 1. Hence, to obtain the true lncRNA-lncRNA pairs, we subtracted the above correlation pairs (*n* = 4,289) to give 18,391,232 lncRNA-lncRNA pairs. lncRNA-lncRNA pairs were divided into two groups (a) *cis* pairs (lncRNAs that lie 500 kb within upstream and downstream of the other lncRNA coordinates) and (b) *trans* (lncRNAs that lie >500 kb upstream and downstream of the other lncRNA coordinates). A total of 3,386 lncRNA-lncRNA *cis* pairs were obtained that were significantly correlating (*p* value < 0.05). However, this included correlation between lncRNA pairs twice (e.g.: A vs B and B vs A). Hence, to obtain the unique pairs, only one of the correlation values between lncRNA pairs were considered to obtain 1,693 lncRNA-lncRNA *cis* pairs. Since lncRNA-lncRNA *trans* pairs were large in number (*n* = 18,378,940), we used stringent cut off (*p* value < 0.05 and |r| >0.9). We found 3,216,060 lncRNA-lncRNA *trans* pairs were significantly correlated (*p* value < 0.05). Out of these pairs 8,547 lncRNA-lncRNA *trans* pairs were unique and having absolute Spearman correlation coefficient greater than 0.9 (Supplementary Figure 4).

#### LncRNAs as miRNA hosts

Further, we wanted to identify long non-coding RNAs that might give rise to small non-coding RNAs, that is, micro RNAs. For this; genomic coordinates of deregulated lncRNAs from Arraystar microarray analysis were mapped to the genomic coordinates of the list of 2794 miRNAs downloaded from miRBase (http://www.mirbase.org/ftp.shtml; version 21) [48, 49]. This was performed using “Intersect” function of BEDtools. Several miRNAs and miRNA families were identified; to which differentially expressed lncRNAs in GBM were playing host to- in the form of parent gene. To this we added the miRNA expression data in GBM from TCGA miRNA microarray as well as miRNA expression data from the microarray performed previously for a separate cohort in lab. This enabled us to identify significantly regulated miRNAs in GBM whose parent gene is a deregulated lncRNA in GBM. The regulation of such miRNAs can be thought of being regulated by the parent gene; although further investigation into whether the small non-coding RNA shares its promoter with that of the lncRNA is required.

#### LncRNAs as miRNA decoys

The competing endogenous RNAs (ceRNAs) network was constructed to identify lncRNA-miRNA-mRNA sponge modules by the following step: 1) differentially expressed lncRNAs between GBM and control brain samples from TCGA RNA-seq were taken (*n* = 1,559; fold change ≥ 1.5 & FDR < 0.05). 2) miRcode [50] was used to predict miRNAs that target the differentially expressed lncRNAs, 3) miRNAs obtained from the above analysis was used to obtain their experimentally validated target mRNAs extracted from miRWalk [51].

Out of the differentially regulated lncRNAs (*n* = 1,559), a total of 1,092 lncRNAs were present in miRcode database. From miRcode database, we obtained 26,620 lncRNA-miRNA pairs having expression data in TCGA GBM samples of which 2,531 lncRNA-miRNA pairs (lncRNA *n* = 695; miRNA *n* = 133) showed negative correlation. Next, miRNAs obtained from the above analysis was used to detect their experimentally validated mRNA targets obtained from miRWalk. From this analysis, we obtained 47,887 miRNA-mRNA pairs having expression data in TCGA GBM samples from which 5,336 miRNA-mRNA pairs having negative correlation (miRNAs *n* = 125; mRNA *n* = 3060) were taken for consideration. In the next step, we merged the lncRNA-miRNA pairs (*n* = 2,531) with miRNA-mRNA pairs (*n* = 5,336) to create lncRNA-miRNA-mRNA sponge modules (*n* = 150,684). In the next step, from these sponge modules, we selected only those modules that showed a positive correlation between mRNA-lncRNA and all three (lncRNAs, miRNA and mRNA) showed upregulation in GBM compared to control samples (*n* = 9,448). In the last step, those modules wherein the abundance of lncRNA transcript levels is significantly higher than that of mRNA which resulted in a true lncRNA-miRNA-mRNA sponge module (*n* = 408), wherein the lncRNA is highly likely to work as a sponge such that the target mRNA will be spared for the miRNA. The unique set of mRNAs (*n* = 134) from this finally selected lncRNA-miRNA-mRNA sponge modules were used for DAVID pathway analysis to find out important pathways that are regulated by these sponge modules.

### LncRNA validation by real-time PCR

The RNA was reverse transcribed using High-Capacity cDNA Reverse transcription kit (Thermo Fisher Scientific). Dynamo SYBR qRT-PCR kit (Finnzymes) was used for real- time PCR assays. The mRNA levels of RPL35 and ATP5G were used as endogenous controls. Specific primer pairs for the lncRNAs were ordered from Sigma Aldrich and their sequences (5’-3’) are as follows - HOXD-AS2: CAAAGGAACTGCTCTGGTGA, CCAAGCTTCTTGTGTCCTCTG; CRNDE: TCATGATTAGCAGGCAGACG, ACAAACGGTCACCACTACCC; RP11- 389G6.3: CAATATGCAGGATGGGAAGG, CCAGAGTCCTTGGAAACCAC; RP11-325I22.2: AATACGGGTTGAGCATCAGG, AATCGCCATCCTTTCACAAC; ANRIL: TTTCCTACGAAGCTGGGTGA, GTAAAACGCAACAAGATAGAGAAGC.

### ANRIL silencing

LN229 and T98G cells were seeded at the density of 0.8 million in 60 mm petri dish. After 16 hours of initial seeding, cells were transfected with 8 μg of pSR_ANRIL and control plasmids using opti-MEM and lipofectamine reagents. After 6 hours of transfection, opti-MEM was replaced by complete DMEM (DMEM+ 10% FBS). Post 48 hours of transfection, cells were harvested, counted and seeded for proliferation and colony forming ability. The knockdown of ANRIL in the transfected cells over the vector control transfected cells was confirmed by qRT- PCR.

### Cell proliferation by MTT

Cells either transfected with pSR_ANRIL or control plasmids were harvested and plated at density of 1500 cells per well in 96 well plate in triplicates. Each day a triplicate set for each condition were treated with 10 μl (5 mg/ml in PBS) of MTT [3-(4,5-Dimethylthiazol-2-yl)-2,5-Diphenyltetrazolium Bromide] to quantify the viable cell number. Three hours after addition of MTT, formazan crystal were dissolved in 200 μl of DMSO, mixed well and absorbance was measured at 570 nm using Elisa plate reader. The statistical significance were calculted by student’s *T* test.

### Cell proliferation by trypan blue

Cells either transfected with pSR_ANRIL or control plasmids were harvested and plated at density of 30000 cells per well in 12 well plate in duplicates. Each day, a duplicate set was harvested, washeshed with PBS, resuspended in 0.32% trypan blue and viable cells were counted using haemocytometer. statistical significance were calculted by student’s *T* test.

### Colony formation assay

Cells either transfected with pSR_ANRIL or control plasmids were harvested and plated at density of 2000 cells per well in 6 well plate in triplicates. Every third day, the culture media were replaced. After 12 days of culturing, the colonies were fixed with 100% chilled methanol for 30 min and then stained with 0.05% crystal violet for 30min and photographed.

## SUPPLEMENTARY MATERIALS FIGURES AND TABLES



























## Abbreviations

lncRNA: long non-coding RNA
ceRNA: competing endogenous RNA
ANRIL: antisense non-coding RNA in the INK4 locus
GBM: Glioblastoma
CNV: Copy Number Variation
TCGA: The Cancer Genome Atlas
miRNA: Micro RNA
HR: Hazard Ratio
G-CIMP: Glioma-CpG island methylator phenotype
