# Supplementary material for: Genetic landscape of long noncoding RNA (lncRNAs) in glioblastoma: identification of complex lncRNA regulatory networks and clinically relevant lncRNAs in glioblastoma
Source: Oncotarget. 2018 Jul 3;9(51):29548–64. doi: 10.18632/oncotarget.25434 (PMC6049862; doi:10.18632/oncotarget.25434)
Supplement: Supplementary file 1 [file oncotarget-09-29548-s001.pdf]

Genetic landscape of long noncoding RNA (lncRNAs) in glioblastoma: identification of complex lncRNA regulatory networks and clinically relevant lncRNAs in glioblastoma

SUPPLEMENTARY MATERIALS

A

lncRNA subtypes

S. no	Arraystar lncRNA classification	Upregulated	Downregulated	Total regulated
1	Intergenic	1462	3258	4720
2	Bidirectional	200	212	412
3	Intronic antisense	291	616	907
4	Intron sense overlapping	113	154	267
5	Natural antisense	394	489	883
6	Exon Sense overlapping	230	214	444
7	Unclassified	84	73	157
Total		2774	5016	7790

B

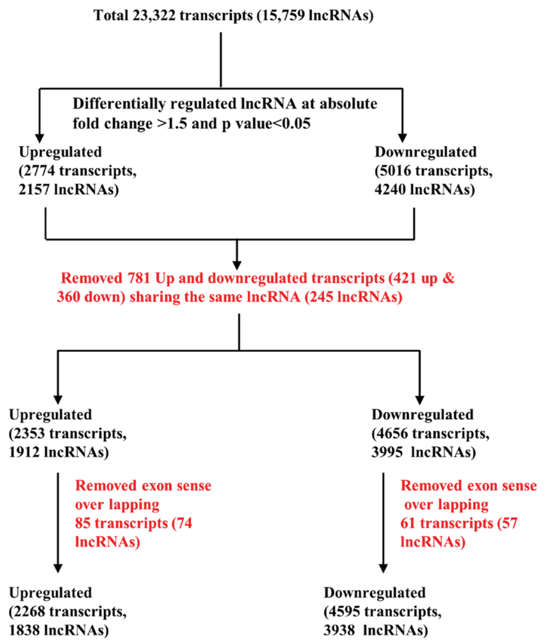

Supplementary Figure 1: Filtering of lncRNAs for further analysis. (**A**) Tabular representation of differentially regulated different types of lncRNA in our GBM cohort as compared to control brain samples. (**B**) Flow chart depicting the strategy employed to filter out lncRNA transcripts. The transcripts of a gene that showed both upregulation and downregulation and exon sense overlapping transcripts were filtered out.

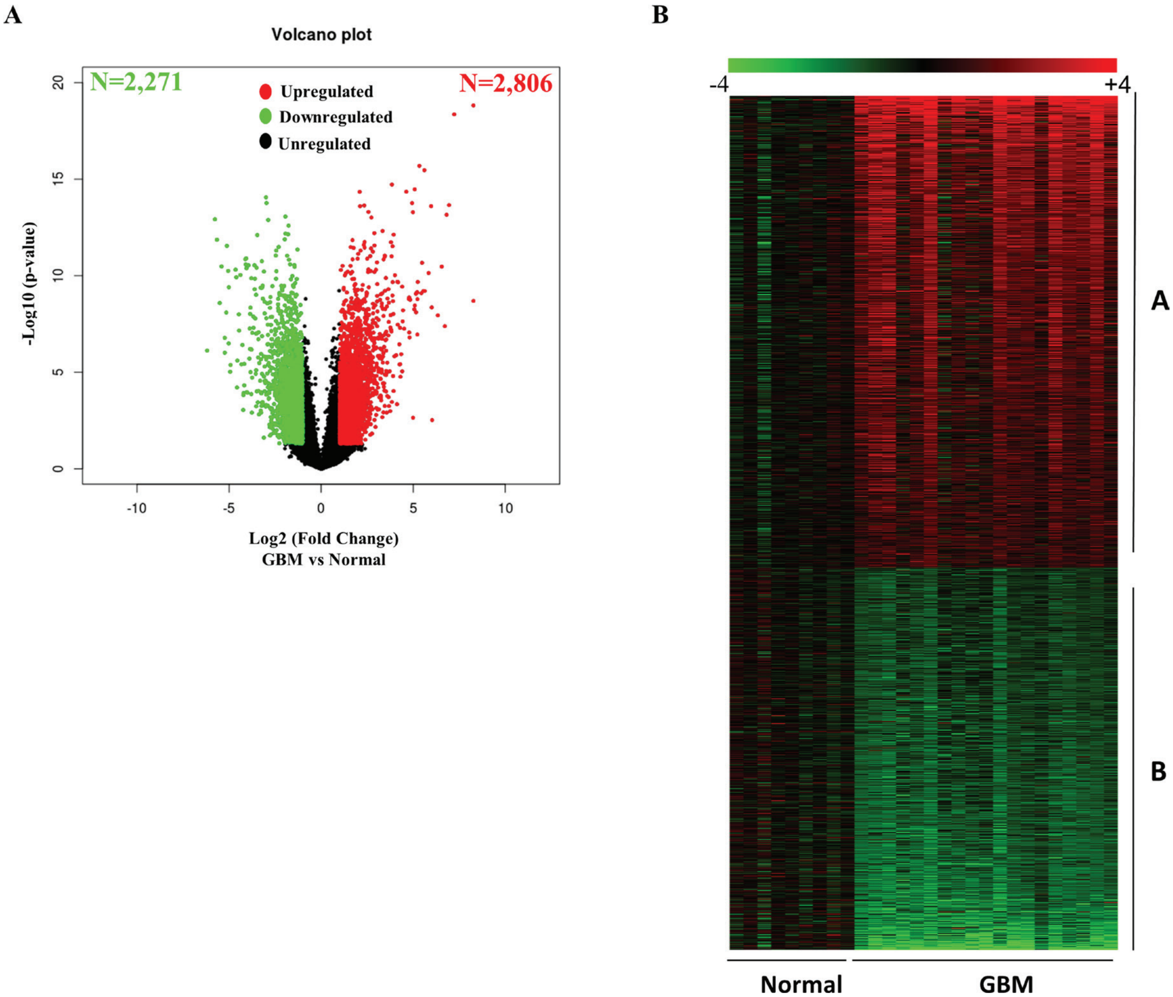

Supplementary Figure 2: mRNA profiling in GBM samples. Dysregulated mRNAs were profiled in the same samples that were subjected to lncRNA microarray analysis. Out of 23,376 mRNA transcripts profiled, 2,806 were upregulated and 2271 were downregulated in GBM tissues as compared to control brain tissue. (**A**) Volcano plot depicting the dysregulated mRNAs, with the red dots as upregulated mRNAs and green dots as downregulated transcripts. (**B**) Heat map of dysregulated mRNAs in GBM tissue over control brain tissue. Upregulated transcripts are denoted in red and downregulated in green.

lncRNA - mRNA correlation

Differentially regulated lncRNA at absolute fold change >2 and p value<0.05

Up regulated lncRNA (1,404)
Down regulated lncRNA (2,885)
Total differentially regulated lncRNA (4,289)

Differentially regulated mRNA at absolute fold change >2 and p value<0.05

Up regulated mRNA (2,806)
Down regulated mRNA (2,270)
Total differentially regulated mRNA (5,076)

lncRNA-mRNA pairs (4,289 X 5,076)= **21,770,964**

lncRNA-mRNA cis pairs = 10,968

lncRNA-mRNA cis significant pairs (p<0.05) = 2,674

Correlation	lncRNA regulation	mRNA regulation	# pairs	#lncRNAs	#mRNAs
Positive	Up	Up	455	290	342
Positive	Down	Down	693	501	506
Negative	Up	Down	165	113	117
Negative	Down	Up	683	454	519
Total			1,996	1,148	1,389

lncRNA-mRNA trans pairs = 21,759,996

lncRNA-mRNA trans significant pairs (p<0.05)= 4,679,124

lncRNA-mRNA trans significant pairs & |r| > 0.9 = 24,631

Correlation	lncRNA regulation	mRNA regulation	# pairs	#lncRNA	#mRNA
Positive	Up	Up	2,613	205	704
Positive	Down	Down	13,732	1,073	1,000
Negative	Up	Down	434	81	141
Negative	Down	Up	6,680	325	563
Total			23,459	1,327	1,872

Supplementary Figure 3: Correlation of lncRNAs with mRNAs. A flow chart showing the strategy used for correlating lncRNAs with mRNAs in our cohort.

lncRNA - lncRNA correlation

Differentially regulated lncRNA at absolute fold change >2 and p value<0.05

Up regulated lncRNA (1,404)
Down regulated lncRNA (2,885)
Total differentially regulated lncRNA (4,289)

Differentially regulated lncRNA at absolute fold change >2 and p value<0.05

Up regulated lncRNA (1,404)
Down regulated lncRNA (2,885)
Total differentially regulated lncRNA (4,289)

lncRNA-lncRNA pairs (4,289 X 4,289) = 18,395,521 – 4,289 = **18,391,232**

lncRNA-lncRNA cis pairs = 12,292

lncRNA–lncRNA cis significant pairs (p<0.05) = 3,386

lncRNA-lncRNA cis unique pairs = 1,693

Correlation	lncRNA1 regulation	lncRNA2 regulation	# pairs	#lncRNA1	#lncRNA2
Positive	Up	Up	549	325	327
Positive	Down	Down	684	499	509
Negative	Up	Down	91	73	66
Negative	Down	Up	58	54	47
Total			1,382	901	921

lncRNA-lncRNARNA trans pairs = 18,378,940

lncRNA-lncRNA trans significant pairs (p<0.05)= 3,216,060

lncRNA-lncRNA trans significant pairs & |r| > 0.9 = 17,094

lncRNA-lncRNA trans unique pairs = 8,547

Correlation	lncRNA1 regulation	lncRNA2 regulation	# pairs	#lncRNA1	#lncRNA2
Positive	Up	Up	359	126	116
Positive	Down	Down	7,292	932	927
Negative	Up	Down	255	41	110
Negative	Down	Up	238	103	34
Total			8,144	1,082	1,070

Supplementary Figure 4: Correlation of lncRNAs with other lncRNAs. A flow chart showing the strategy used for correlating lncRNAs with other lncRNAs in our cohort.

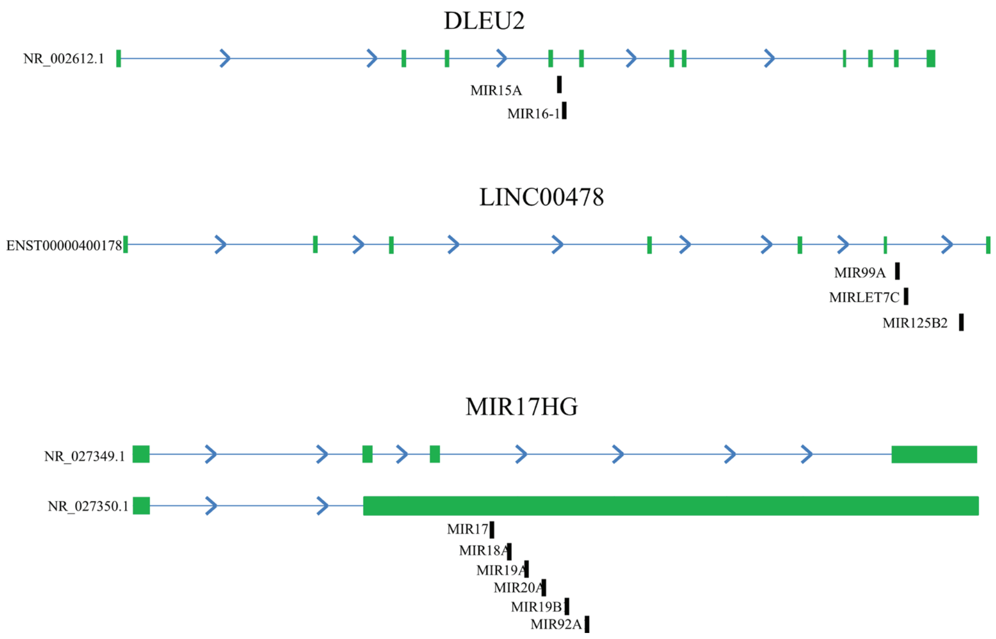

Supplementary Figure 5: Genomic structure of lncRNAs. Schematic representation of the genomic locus of DLEU2, LINC00478 and MIR17HG.

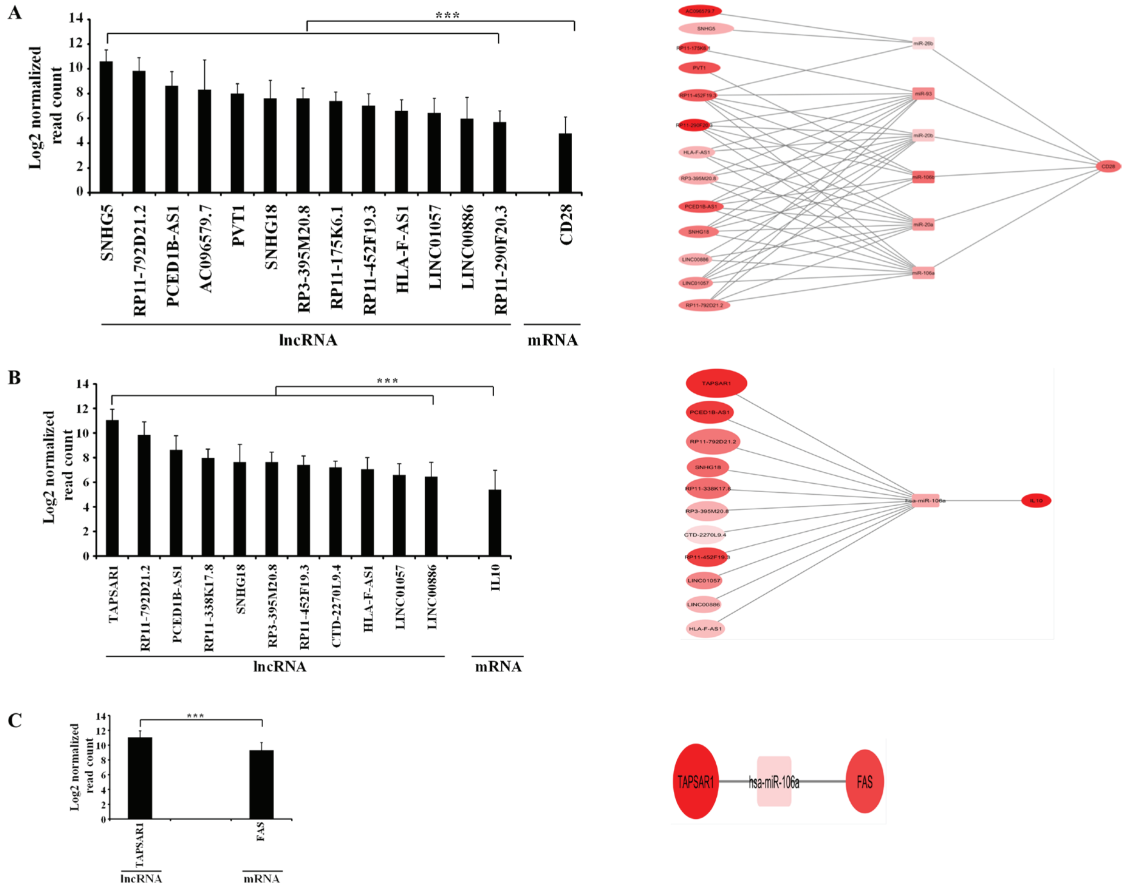

Supplementary Figure 6: ceRNA network of activation of T and B lymphocytes pathway genes in GBM. lncRNA-miRNA-mRNA sponge network for CD28 (**A**), IL10 (**B**) and FAS (**C**) in GBM is shown. Bar graph shows the average log_2 abundance of lncRNA and mRNA sponge network molecules in GBM samples with standard deviation. Square node represents miRNA and circle nodes represent lncRNAs and mRNA. The Size of the node (lncRNA and mRNA) is proportional to average abundance of the molecule in GBM samples. Intensity of colour represents the log_2 fold change between GBM samples and control brain samples.

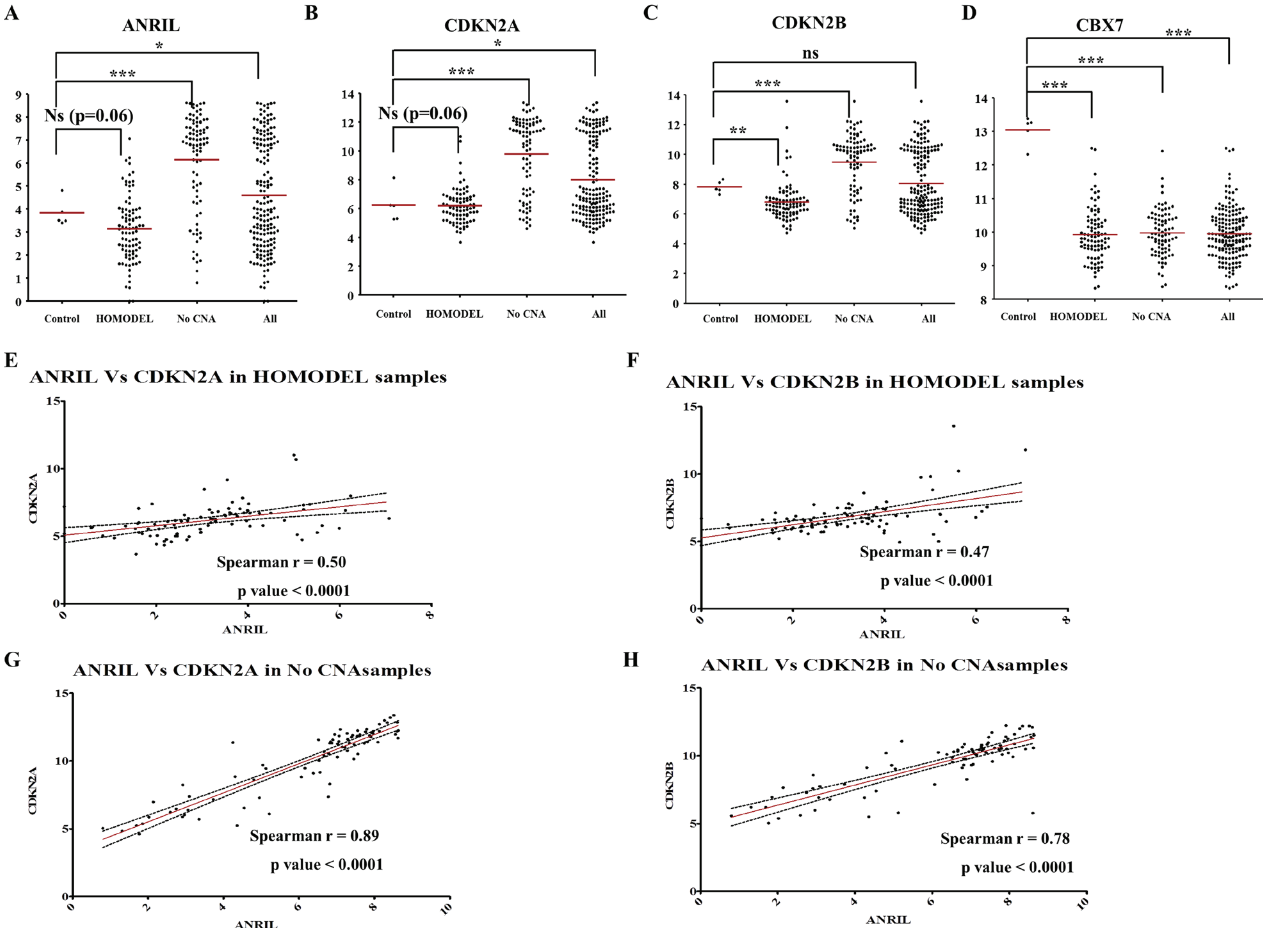

Supplementary Figure 7: Regulation and correlation of ANRIL. mRNA expression of ANRIL (**A**) CDKN2A (**B**), CDKN2B (**C**) and CBX7 (**D**) in control brain (*n* = 5); CDKN2A/2B homozygous deleted samples (*n* = 90); no copy number alteration in CDKN2A/2B locus samples (*n* = 82) and all GBM samples (*n* = 172) from TCGA RNA sequencing cohort. (**E**) Correlation between ANRIL and CDKN2A in CDKN2A/2B homozygous deleted samples (*n* = 90) in TCGA RNA sequencing cohort. (**F**) Correlation between ANRIL and CDKN2B in CDKN2A/2B homozygous deleted samples (*n* = 90) in TCGA RNA sequencing cohort. (**G**) Correlation between ANRIL and CDKN2A in no copy number alteration in CDKN2A/2B samples (*n* = 82) in TCGA RNA sequencing cohort. (**H**) Correlation between ANRIL and CDKN2A in no copy number alteration in CDKN2A/2B samples (*n* = 82) in TCGA RNA sequencing cohort.

Supplementary Table 1: List of samples used in this study for microarray analysis

S. no.	Sample_Id	"Sample type/Grade "	"#450 K methylation"	$GSE79122	Gender	"Age (years)"
1	G1	GBM	methy	GSM2086371	male	63
2	G2	GBM	methy	GSM2086372	male	45
3	G3	GBM	methy	GSM2086373	female	61
4	G4	GBM	methy	GSM2086396	male	54
5	G5	GBM	methy	GSM2086397	male	51
6	G6	GBM	methy	GSM2086398	male	55
7	G7	GBM	methy	GSM2086400	male	62
8	G8	GBM	methy	GSM2086379	male	20
9	G9	GBM	methy	GSM2086380	male	34
10	G10	GBM	methy	GSM2086384	male	50
11	G11	GBM	methy	GSM2086377	male	50
12	G12	GBM	methy	GSM2086386	female	55
13	G13	GBM	methy	GSM2086385	male	50
14	G14	GBM	methy	GSM2086370	male	45
15	G15	GBM	methy	GSM2086374	male	60
16	G16	GBM	methy	GSM2086378	male	65
17	G17	GBM	methy	GSM2086376	male	37
18	G18	GBM	methy	GSM2086383	male	45
19	G19	GBM	methy	GSM2086387	female	45
20	G20	GBM	methy	GSM2086388	femlae	40
21	G21	GBM	methy	GSM2086366	male	56
22	G22	GBM	methy	GSM2086392	female	49
23	N1	Control Brain	methy	GSM2086323	male	25
24	N2	Control Brain	ND	NA	NA	NA
25	N3	Control Brain	ND	NA	NA	NA
26	N4	Control Brain	ND	NA	NA	NA
27	N5	Control Brain	methy	GSM2086325	female	51
28	N6	Control Brain	methy	GSM2086326	female	33
29	N7	Control Brain	ND	NA	NA	NA
30	N8	Control Brain	methy	GSM2086324	female	15

#Integrative functional genomic analysis identifies epigenetically regulated fibromodulin as an essential gene for glioma cell migration.Mondal B, Patil V, Shwetha SD, Sravani K, Hegde AS,Arivazhagan A, Santosh V, Kanduri M, Somasundaram K.Oncogene. 2017; 36:71–83.
$ID as per geo GSE79122 (https://www.ncbi.nlm.nih.gov/geo/query/acc.cgi?acc=GSE79122).
ND- Not done.
NA- Not available.

Supplementary Table 2: Differentially regulated lncRNAs between GBM vs control brain samples in our cohort. See Supplementary_Table_2

Supplementary Table 3: Differentially regulated mRNAs between GBM vs control brain samples in our cohort. See Supplementary_Table_3

Supplementary Table 4: Correlation between lncRNAs and mRNAs in cis. See Supplementary_Table_4

Supplementary Table 5: Correlation between lncRNAs and mRNAs in trans. See Supplementary_Table_5

Supplementary Table 6: Enriched gene ontology and pathways in cis. See Supplementary_Table_6

Supplementary Table 7: Enriched gene ontology and pathways in trans. See Supplementary_Table_7

Supplementary Table 8: Correlation between lncRNAs and lncRNAs in cis. See Supplementary_Table_8

Supplementary Table 9: Correlation between lncRNAs and lncRNAs in trans. See Supplementary_Table_9

Supplementary Table 10: List of lncRNA genes that behave as hosts or parent genes for miRNAs and miRNA families. See Supplementary_Table_10

Supplementary Table 11: Competing endogenous RNAs (ceRNA) network in GBM (TCGA RNA-Seq). See Supplementary_Table_11

Supplementary Table 12: List of enriched pathways in competing endogenous RNAs (ceRNA) network in GBM (TCGA RNA sequencing). See Supplementary_Table_12

Supplementary Table 13: List of lncRNAs correlating with survival in GBM (TCGA RNA-Seq). See Supplementary_Table_13
